# Display of a Maize cDNA library on baculovirus infected insect cells

**DOI:** 10.1186/1472-6750-8-64

**Published:** 2008-08-12

**Authors:** Helene Y Meller Harel, Veronique Fontaine, Hongying Chen, Ian M Jones, Paul A Millner

**Affiliations:** 1 Faculty of biological sciences, University of Leeds, Leeds, LS2 9JT, UK; 2 UMR INRA/USTL 1281, Stress Abiotiques et Différenciation des Végétaux cultivés 2, Chaussée Brunehaut, Estrées-Mons BP 50136, 80203 Péronne cedex, France; 3 School of Biological Sciences, University of Reading, Whiteknights, Reading, Berks, RG6 6AJ, UK

## Abstract

**Background:**

Maize is a good model system for cereal crop genetics and development because of its rich genetic heritage and well-characterized morphology. The sequencing of its genome is well advanced, and new technologies for efficient proteomic analysis are needed. Baculovirus expression systems have been used for the last twenty years to express in insect cells a wide variety of eukaryotic proteins that require complex folding or extensive posttranslational modification. More recently, baculovirus display technologies based on the expression of foreign sequences on the surface of *Autographa californica *(AcMNPV) have been developed. We investigated the potential of a display methodology for a cDNA library of maize young seedlings.

**Results:**

We constructed a full-length cDNA library of young maize etiolated seedlings in the transfer vector pAcTMVSVG. The library contained a total of 2.5 × 10^5 ^independent clones. Expression of two known maize proteins, calreticulin and auxin binding protein (ABP1), was shown by western blot analysis of protein extracts from insect cells infected with the cDNA library. Display of the two proteins in infected insect cells was shown by selective biopanning using magnetic cell sorting and demonstrated proof of concept that the baculovirus maize cDNA display library could be used to identify and isolate proteins.

**Conclusion:**

The maize cDNA library constructed in this study relies on the novel technology of baculovirus display and is unique in currently published cDNA libraries.

Produced to demonstrate proof of principle, it opens the way for the development of a eukaryotic *in vivo *display tool which would be ideally suited for rapid screening of the maize proteome for binding partners, such as proteins involved in hormone regulation or defence.

## Background

With its rich genetic heritage and well-characterized morphology, *Zea mays *(L. ssp.*mays*) is a very important crop and a model system for cereal genetics and development. Its moderately large genome (>2.5 Mb) comprises up to 80% of repeated retrotransposons [1]. It is in the process of being completely sequenced and is the subject of many recent studies [2-4]. In particular, the maize transcriptome provides a source of new functionally important genes and has been recently studied with various methods such as comparisons between sense and antisense transcriptome [5], serial analysis of gene expression [6], and the construction of PCR-generated, normalized, or subtracted cDNA libraries [7-9].

Display technologies are powerful tools for proteomics studies since they allow direct coupling of proteins with their DNA encoding sequences. The prokaryotic phage display technology is the method of choice when studying non complex proteins but presents significant limitations in the case where folding, post-translational modification or oligomerization of the gene products are needed. Baculovirus surface display is a relatively recent technology, which allows correct expression and presentation of eukaryotic proteins [10]. It is based on the use of baculovirus *Autographa californica *multiple nuclear polyhydrosis virus (AcMNPV) as a vector for expression of a recombinant target sequence on the surface of the virions or the host *Spodoptera frugiperda *(Sf9) cells [11]. The major baculoviral envelope membrane protein, gp64, is usually used as the fusion partner for functional display of a foreign protein or peptide sequence on the viral surface. However, heterologous viral glycoproteins have been shown to be good alternatives to gp64. Such is the case of a truncated form of the vesicular stomatitis virus G glycoprotein (VSV-G) that allows an enhanced level of display as well as alternate presentation of epitopes [12]. Refining the display capabilities of AcMNPV has been the subject of many studies [10,13]. They have led to the development of tools for important applications, such as antigens display for production of antibodies [14], molecular screening and drug delivery into mammalian target cells [15], as well as baculovirus display of peptide libraries forselection of monoclonal antibody epitopes and to identify peptide antigen mimotopes [16,17]. To date however, the only study where baculovirus-based cDNA library were generated did not use baculovirus-infected cells as a display platform [18]. We describe here the construction and preliminary analysis of a baculovirus display cDNA library of maize young etiolated seedlings.

## Results and discussion

### Construction of a maize cDNA display library

Total RNA from young maize etiolated seedlings was reverse transcribed, converted to double stranded cDNA and cloned into the baculovirus transfer vector pAcTMVSVG. This vector, combining the signal peptide of the single major envelope glycoprotein gp64 from AcMNPV with the cytoplasmic and transmembrane domains of VSV-G vesicular stomatitis virus G glycoprotein, allows a high level of displayed proteins as well as alternate presentation of epitopes independent of gp64 [12]. The estimated size of the plasmid library was 2.5 × 10^5 ^independent clones, with a size distribution of cDNA insert ranging from 0.4 to 2 kb.

The resulting library of plasmids constructed in the transfer vector was incorporated into the baculovirus genome by homologous recombination with a modified viral DNA, BAC10:KO_1629_. This viral DNA cannot produce virus without undergoing recombination resulting in efficient transfer of the complexity of the vector library into the baculovirus background [19]. Following transfection of a mixture of linearised viral DNA with multiple aliquots of the transfer vector recombinant library into Sf9 insect cells, virus was harvested at 3 days post infection and the supernatant pooled to form a master virus library. The library was not grown further to avoid degeneration but was stored frozen at -80°C.

### Expression of calreticulin and ABP1

**Figure 1:**
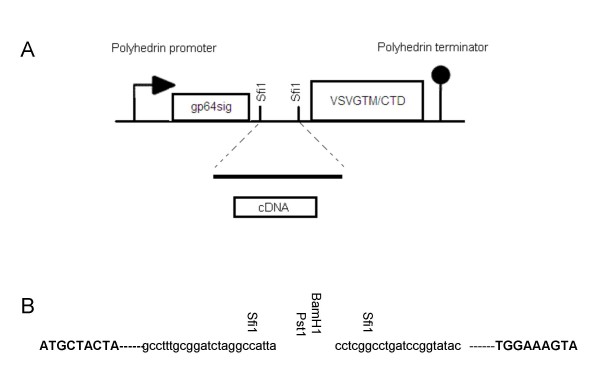
**Construction of the display library**. cDNA fragments were cloned in *Sfi1 *sites of pAcTMVSVG vector providing N-terminal fusion with the gp64 signal peptide and C-terminal fusion with the transmembrane (TM) and cytoplasmic terminal domains (CTD) of the VSV G protein. The chimeric genes were expressed under the control of polyhedrin promoter and terminator. **A**, Schematic representation of baculovirus vector pAcTMVSVG. **B**, Details of the cloning site with, in bold, the start and stop sequences from gp64 and VSV G respectively, in lower case, the sequences of the vector around the *Sfi1 *cloning site, in vertical-orientation, the restriction sites in the multiple cloning site.

A principal of the system is that any valid cDNA sequence from the maize genome would be expressed as a recombinant protein with its N-terminus fused to the gp64 signal sequence and C-terminus fused to the trans-membrane and cytoplasmic domain of the VSV G glycoprotein (Figure 1). Therefore, in order to assess the functionality of the cDNA library, we studied the expression of two well-characterized maize proteins, calreticulin and ABP1.

Calreticulin is a protein abundant in the endoplasmic reticulum (ER) of many eukaryotes, and in particular of maize [20]. ABP1 is an auxin receptor found ubiquitously in vascular plants and originally identified in maize (*Zea mays*) [21] as a soluble protein, a proportion of which is present on the outer surface of the cell [22].

The presence of maize calreticulin and ABP1 cDNAs in the maize seedlings cDNA library was shown directly by PCR on the plasmid library prepared, using primers specific to calreticulin and ABP1 sequences which revealed products of the expected sizes (670 bp and 490 bp respectively, results not shown).

**Figure 2:**
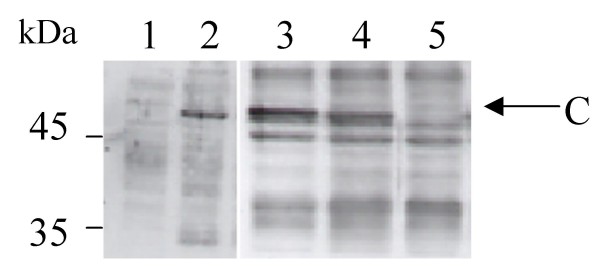
**Expression and display of calreticulin in Sf9 cells infected by the cDNA library**. Crude cell extracts (10 μg of proteins) from non-infected Sf9 cells (lane 1), infected cells before panning (lane 2, 3), positive sorted cells after panning (lane 4) or negative sorted cells after panning (lane 5) were run on a SDS-PAGE (12%) and analysed by Western Blot against maize (lane 1, 2) or tobacco anti-calreticulin antiserum, (lane 3, 4,5). Position of size marker is indicated on the left. Arrow points to calreticulin (C) band.

**Figure 3:**
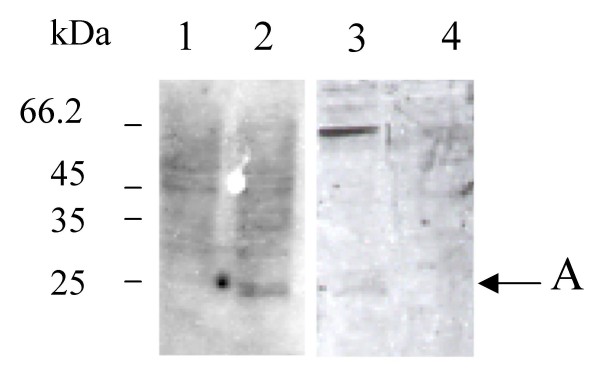
**Expression and display of ABP1 in Sf9 cells infected by the cDNA library**. Crude cell extracts (10 μg of proteins) from non-infected Sf9 cells (lane 1), infected Sf9 cells before panning (lane 2), positive sorted cells after panning (lane 3), or negative sorted cells after panning (lane 4) were run on a SDS-PAGE (12%) and analysed by Western Blot against maize anti-abp1 antiserum. Position of size marker is indicated on the left. Arrow points to ABP1 (A) band.

The correct expression of calreticulin and ABP1 in insect cells infected with the baculovirus stock of the total cDNA library was studied by immunoblotting. On the blots probed with polyclonal antiserum raised against maize calreticulin [20] or maize ABP1 [23], polypeptides were detected in the infected extracts (Figure 2, lane 2 and Figure 3 lane 2) but not in the extract from non-infected cells (Figure 2, lane 1 and Figure 3, lane 2). The polypeptides migrated to a molecular weight corresponding to maize calreticulin (48 kDa) and to glycosylated ABP1 (22 kDa), respectively. The clear expression of glycosylated ABP1 in our system is consistent with previous work showing expression of an active ABP1 from its cDNA in a baculovirus system [24]. It was one of the first examples illustrating that insect cells were appropriate hosts for the expression of plant genes: ABP1 was correctly processed and glycosylated in the endo-membrane system of insect cells. In the case of calreticulin, expression in a baculovirus system of a plant calreticulin is shown here for the first time in this study, although human calreticulin has been co-expressed in a baculovirus system by others [25].

### Display of calreticulin and ABP1

Similarly to what was shown with eGFP expression using pAcTMVSVG vector [12], we expected the expressed proteins to complete the secretory pathway and be retained on the infected cell surface. In order to isolate such cells, we used the magnetic cell sorting (MACS) technology [26]. The indirect magnetic cell labelling was conducted using a primary antibody directed against the protein of interest and anti-immunoglobulin coated beads. MACS technology has been already used for selection of baculovirus-infected cells vs. non-infected ones [27]. In our case however, magnetic cell sorting is used for the biopanning of specific infected cells according to the expression of a surface protein, calreticulin or ABP1, interacting with a specific ligand, the primary antibody. The high conservation of calreticulin sequence in plants allowed us to use an antibody directed against tobacco calreticulin [28]. It showed good recognition of the maize recombinant calreticulin (Figure 2, lane 3) and was used to magnetically label cells displaying calreticulin. The maize ABP1 polyclonal antiserum described before was used to magnetically label cells displaying ABP1.

Virus from positively and negatively biopanned cells were amplified and used to infect fresh insect cells. Expression of the proteins was studied by immunoblotting, as previously described. Maize calreticulin is clearly revealed as a strong band in protein extracts from magnetically labelled cells (Figure 2, lane 4), but cannot be detected in protein extracts from non-labelled cells (Figure 2, lane 5), as expected. Maize ABP1 is also specifically detected, although as a minor band, in labelled cell extracts but not in non-labelled extracts (Figure 3 lane 3 and 4). The weak signal obtained could result from a partial display of the recombinant protein or from a weak recognition by the first antibody because of unspecific interaction with the second antibody. The strong band visible in lane 4 could correspond to a cross-reaction between the first and the second antibody, as was noted in a study showing ABP1 immunoprecipitation [24].

We conclude therefore that calreticulin and ABP1 are expressed and displayed by insect cells infected by baculovirus containing the whole maize cDNA, demonstrating the proof of principle that our novel maize cDNA display library constructed using recombinant baculoviruses can be used to isolate proteins.

## Conclusion

We have constructed a maize cDNA display library in the baculovirus transfer vector pAcTMVSVG, and have screened it by magnetic cell sorting for the expression and the display of two known proteins, calreticulin and ABP1.

Both proteins are moderately abundant in maize, their expression being inducible by various developmental stage or stimuli [20,22,29]. The method of detection by magnetic cell sorting was sensitive enough to reveal their presence on insect cells infected by the cDNA library. Based on the ease of identification, we would expect to detect proteins encoded by less abundant mRNAs as well, especially after normalization and/or subtraction of the amplified library [30].

The library described here is based on baculovirus-infected cells as a display platform, and not only as an expression system as it is the case in the only other published baculovirus-based cDNA expression library [18]. For this reason, it is unique in currently published cDNA library. In addition, it is also, to our knowledge, the first cDNA display library generated from *Zea mays*.

The presented study advances the development and use of cDNA display libraries expressed in baculovirus that could enhance gene discovery. In particular, over 2 million maize cDNA sequences are now available [2] which could be a source for discovery of new encoded proteins using this new eukaryotic *in vivo *display tool.

Fluorescence activated cell sorting (FACS) is the classical methodology used for selection of molecules in a baculovirus surface display, as in the case of displayed peptide sequences [14]. Here we report the use of MACS for an efficient biopanning of baculovirus-infected insect cell displaying a desired protein and therefore binding to a specific antibody. The same principle of methodology could be very useful in addressing proteomic questions, such as rapid screening of the maize proteome for binding partners, for example proteins involved in hormone regulation or defence. This eukaryotic *in vivo *display tool would ultimately allow biopanning of a cDNA library displayed on the virus surface with a target ligand of interest, thus allowing rapid screening of a proteome for binding partners.

## Methods

### Library construction in the pACMNPVTM vector

Total RNA (1.5 μg) was extracted from etiolated 5 days old maize seedlings grown under dark conditions using the RNeasy Plant Mini Kit (Qiagen Ltd., UK). After DNase treatment (DNA-free™ kit from Ambion Applied Biosystem, UK), mRNA was isolated using the Fast Track2 kit (Invitrogen Ltd., UK). Double stranded cDNA was synthesised using the Smart ™ cDNA library construction kit (Clontech UK): single-stranded cDNAs were obtained by reverse transcription, tagged by the SMART IV oligonucleotide, which allow template switching and synthesis of second strand by the reverse transcriptase. Subsequently, the double stranded cDNA were enriched by Long Distance PCR (LD PCR). The PCR amplification products were subjected to *Sfi*I digestion and size fractionation. cDNA *Sfi*I fragments of 0.2 to 4 kb were then ligated to pACVSVGTM vector linearised with *Sfi*I. Test ligations conducted with 20 ng of vector and 20–50 ng of cDNA were electroporated into *Escherichia coli*. Optimal ratio of cDNA to vector allowing size distribution of cDNA inserts from 0.4 to 2 kb was detected by colony PCR using the primers pACsfiIfw: 5'-gcctttgcggatctaggccatta-3' and pACsfiIrev:5'-gtataccggatcaggccgagg-3' (Invitrogen Ltd., UK) and Biotaq Red DNA polymerase (Bioline Ltd., UK). The selected ligations were transformed into SURE2 supercompetent *Escherichia coli *(Stratagene, UK), or electro-transformed into *E. coli *DH5α according to the manufacturer's protocol (Bio-Rad, UK). The number of independent bacterial clones obtained were quantified to estimate the size of the library, and plasmid midi-preps (Qiagen Ltd., UK) were prepared and pooled together.

### Transfection to Insect cells

Insect *S. frugiperda *(Sf9) cells were plated at 5 × 10^5 ^cells/well in a six-well plate and allowed to attach for at least 30 minutes. After one wash, the cells were resuspended in 1 ml of serum-free medium SF-900 II SFM (Invitrogen Ltd., UK). For each transfection, 0.1 μg of linearised BAC10:KO_1629 _DNA was mixed with 0.5 μg of cDNA plasmids library in a total volume of 12 μl. Lipofectin (Invitrogen Ltd., UK) (8 μl) was diluted with 4 μl of water and then mixed with the DNA mixture. Following an incubation for 15-min at room temperature, the DNA-lipofectin complexes were added to the cell monolayers and incubated overnight at 28°C. The cells were then washed with fresh SF-900 II SFM medium and incubated for 4 days at 28°C. The culture was centrifuged to remove cells and the supernatant was kept at 4°C. The virus stock was titrated by plaque assays by absorbing serial dilutions of the viral preparation for 1 to 2 hours onto 2 ml of Sf9 cells 50% confluent in 6-well-plates. After incubation, viruses were removed and 2 ml of 1% agar (w/v) in SF-900 II SFM medium were added to each well and left to solidify. SF-900 II SFM medium (1 ml) was then added to each well and the plates were incubated at 28°C for 3 to 4 days. At the end of the incubation the top medium was removed and the cells were stained with Neutral red (1/13 dilution of 0.33% (v/v) stock solution) for 1 to 2 hours, and the plaques were counted after 3 hours.

### Magnetic biopanning of the library

The maize cDNA library was biopanned using the magnetic sorting technology (Miltenyi Biotec Ltd., UK). Insect cells (10^8^) were infected with the virus stock (MOI of 1 pfu/cell), harvested at 2 days post-infection, and resuspended in 1 ml of buffer PBS pH7.2 (137 mM NaCl, 2.7 mM KCl, 8 mM Na_2_HPO_4_, 2 mM KH_2_PO_4_) supplemented with 0.5% BSA (w/v) and 2 mM EDTA. Cells were then labelled for 5 minutes on ice with primary antibody previously incubated for 60 minutes at 28°C with non-infected cells. Cells were washed twice, resuspended in 1 ml buffer, and subsequently coated with anti-rabbit magnetic beads for 15 minutes at 4°C. After being washed and resuspended in 1 ml buffer, cells were passed through pre-separation filter and subjected to magnetic separation through a cell-separation MS column ((Miltenyi Biotec Ltd., UK) according to the manufacturer protocol. Magnetically labelled cells bound to the column were flushed out with 2 to 3 ml of SF-900 II SFM medium. Both the non-labelled cells coming in the flowthrough, and the labelled cells were incubated at 28°C for one day in SF900 II SFM serum-free medium. Virus stocks were then amplified for 4 days, and stored at -20°C in SF-900 II SFM medium with 2% serum (w/v) for further studies.

### SDS-PAGE and Immunoblotting

Insect cells (10^6^/ml) were infected with virus stocks for 3 days (MOI of 1 pfu/cell) in 6 wells plates and protein extracts from cell pellets were collected by resuspending pellets in PBS pH 7.4 and heating for 5 minutes at 85°C in SDS sample buffer (100 mM Tris pH6.9, 4% SDS (w/v), 20% Glycerol (v/v), 0.1% Bromophenol Blue (v/v), 250 mM DTT). Protein contents were then resolved by 12% SDS-PAGE with a 14.4–116 kDa molecular weight marker (Fermentas, UK) and electrotransferred to nitrocellulose membrane using a Trans-Blot SD semi-dry electrophoretic transfer cell (Bio-Rad, UK). After being washed, the membrane was blocked with 5% milk (w/v) powder in PBST (PBS containing 0.1% Tween20), and incubated with antibodies raised against maize or tobacco proteins diluted 10^-4^in PBST at room temperature for 1 h. Polyclonal serum against maize proteins, calreticulin and ABP1, and tobacco calreticulin were kindly supplied by Prof. R. Napier (HRI, UK) and Dr. J. Denecke (University of Leeds, UK), respectively. After six washes with PBST, the membrane was then incubated with anti-rabbit IgG HRP-linked whole antibody (Amersham plc, UK) diluted 10^-3^, washed, and detected by ECL detection (Amersham plc, UK), as recommended by the manufacturer.

## Authors' contributions

HYMH carried out most of the practical work of the study and the redaction of the manuscript. VF carried out the synthesis of double-strand cDNA from maize RNA, and their digestion. HC and IMJ conceived the technological approach of the study and guided the transfection and biopaning steps. IMJ and PAM helped to draft the manuscript. PAM initiated the concept of the study, participated in its design and coordination. All authors read and approved the final manuscript.
